# Modulation of Pathological Pain by Epidermal Growth Factor Receptor

**DOI:** 10.3389/fphar.2021.642820

**Published:** 2021-05-12

**Authors:** Jazlyn P. Borges, Katrina Mekhail, Gregory D. Fairn, Costin N. Antonescu, Benjamin E. Steinberg

**Affiliations:** ^1^ Neurosciences and Mental Health Program, The Hospital for Sick Children, Toronto, ON, Canada; ^2^ Department of Physiology, University of Toronto, Toronto, ON, Canada; ^3^ Keenan Research Centre for Biomedical Science, St. Michael’s Hospital, Toronto, ON, Canada; ^4^ Department of Biochemistry, University of Toronto, Toronto, ON, Canada; ^5^ Department of Surgery, University of Toronto, Toronto, ON, Canada; ^6^ Department of Laboratory Medicine and Pathobiology, University of Toronto, Toronto, ON, Canada; ^7^ Department of Chemistry and Biology, Ryerson University, Toronto, ON, Canada; ^8^ Department of Anesthesia and Pain Medicine, The Hospital for Sick Children, Toronto, ON, Canada

**Keywords:** epidermai growth factor receptor, neuropathic pain, animal models, inflammation, membrane traffic, receptor tyrosine kinase

## Abstract

Chronic pain has been widely recognized as a major public health problem that impacts multiple aspects of patient quality of life. Unfortunately, chronic pain is often resistant to conventional analgesics, which are further limited by their various side effects. New therapeutic strategies and targets are needed to better serve the millions of people suffering from this devastating disease. To this end, recent clinical and preclinical studies have implicated the epidermal growth factor receptor signaling pathway in chronic pain states. EGFR is one of four members of the ErbB family of receptor tyrosine kinases that have key roles in development and the progression of many cancers. EGFR functions by activating many intracellular signaling pathways following binding of various ligands to the receptor. Several of these signaling pathways, such as phosphatidylinositol 3-kinase, are known mediators of pain. EGFR inhibitors are known for their use as cancer therapeutics but given recent evidence in pilot clinical and preclinical investigations, may have clinical use for treating chronic pain. Here, we review the clinical and preclinical evidence implicating EGFR in pathological pain states and provide an overview of EGFR signaling highlighting how EGFR and its ligands drive pain hypersensitivity and interact with important pain pathways such as the opioid system.

## Introduction

Chronic pain is an international health priority (Kamerman et al., 2015), with a prevalence approaching 30% in North American adults (Moulin et al., 2002; Johannes et al., 2010). In addition to the physical and psychological toll chronic pain places on patients and their families, its societal and economic strain are unparalleled (Gaskin and Richard, 2012). A significant proportion of patients with chronic pain suffer from neuropathic pain (Yawn et al., 2009). Neuropathic pain is defined as pain that arises from a lesion or disease of the somatosensory nervous system (International Association for the Study of Pain, 2017). Clinically, neuropathic pain is characterized by spontaneous pain, increased response to noxious stimuli (hyperalgesia), and the perception of pain from innocuous stimuli (mechanical allodynia) (Baron, 2006). Many clinical pathologies, such as cancer, trauma, and diabetes, can result in neuropathic pain development (Kamerman et al., 2015; Bouhassira and Attal, 2019; Edwards et al., 2019). Unfortunately, neuropathic pain is often resistant to standard analgesics, which are further limited by a variety of adverse side effects (Colloca et al., 2017; Cavalli et al., 2019). As such, a better understanding of the molecular mechanisms of pain hypersensitivity is greatly needed to identify new therapeutic targets and novel treatments for neuropathic pain patients.

Growth factors and their cognate receptors promote pain sensitization and have been identified as therapeutic targets in the treatment of neuropathic pain (Chang et al., 2016). These include nerve growth factor (NGF) (Chang et al., 2016), brain-derived neurotrophic factor (Chen et al., 2014), platelet-derived growth factor (Narita et al., 2005; Donica et al., 2014; Xu et al., 2016; Barkai et al., 2019) and insulin-like growth factor 1 (Wang et al., 2014; Forster et al., 2019). The receptors of these growth factors belong to the receptor tyrosine kinase (RTK) family. More recently another RTK, the epidermal growth factor receptor (EGFR), has been identified as a potential therapeutic target for neuropathic pain. EGFR has crucial roles in prenatal development and adult tissue homeostasis, as signaling by the active receptor controls a wide array of cellular functions including growth, proliferation, metabolism and survival (Miettinen et al., 1995; Sibilia and Wagner, 1995; Threadgill et al., 1995; Sibilia et al., 1998). Thus, aberrant signaling by EGFR drives tumorigenesis and the progression of many cancers (Sigismund et al., 2018). Various therapeutics have been designed to target EGFR in cancer treatments, including antibody therapeutics such as cetuximab, and small-molecule tyrosine kinase inhibitors such as gefitinib and erlotinib (Lemmon et al., 2014).

Here, we first review the clinical and genetic evidence implicating EGFR in pain, emphasizing neuropathic pain, which has been the primary focus of recent studies. We next provide an overview of EGFR signaling, highlighting possible mechanisms by which EGFR and its ligands may influence pain hypersensitivity and modulate inflammatory mediators of pain. We propose future studies directed at better understanding the role of the EGFR family in pain signalling in order to allow evaluation of whether EGFR-related therapeutics may be repositioned for the treatment of neuropathic pain.

### Clinical Data and Genetic Links

Pain begins with the detection of noxious stimuli by specialized peripheral sensory neurons, termed nociceptors (Julius and Basbaum, 2001; Basbaum et al., 2009; Baral et al., 2019). These fibers innervate tissues and organs and have cell bodies located within the dorsal root and trigeminal ganglia. Following stimulation such as extremes in temperature, mechanical injury and injurious chemicals, nociceptors transduce electrical signals that travel to the dorsal horn of the spinal cord, where nociceptors synapse with second-order neurons that carry signals to higher central nervous system relay centres. Structural lesions or disease processes that affect signaling along these somatosensory pathways can result in neuropathic pain. The resulting somatosensory dysfunction can lead to spontaneous pain, allodynia and hypersensitivity (Scholz et al., 2019). For example, cancer can result in direct damage to the nervous system through a primary tumor or a metastatic process or can indirectly trigger chemotherapy-induced neuropathy (Bennett et al., 2019).

**FIGURE 1:**
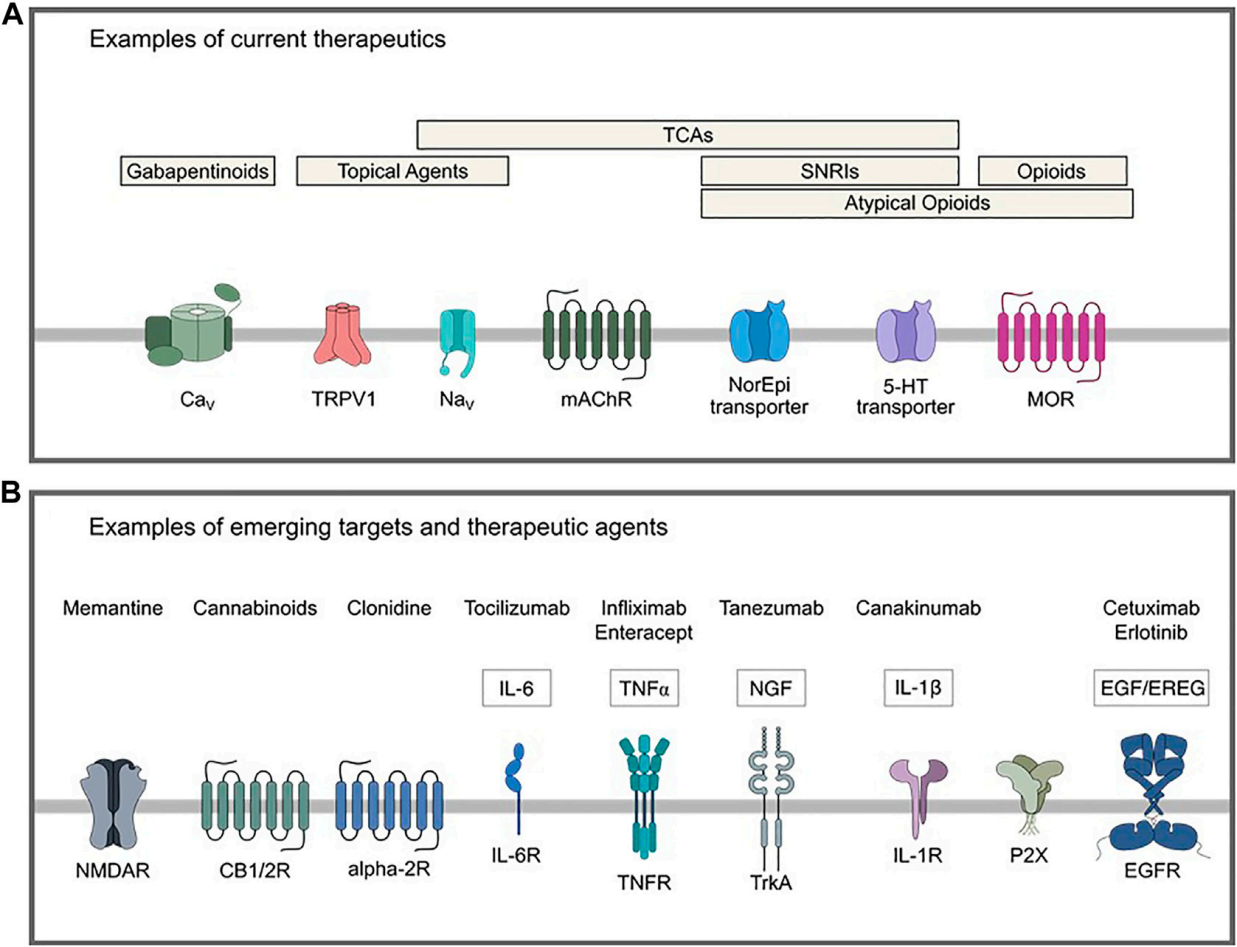
Examples of current and emerging potential therapeutics for treatment of neuropathic pain. **(A)** Current therapeutics include gabapentinoids that act on the α2δ-1 calcium channel subunit, topical agents with targets such as TRPV1 or voltage-gated sodium channels, opioids, atypical opioids (mixed/partial agonists, some of which have actions as an SNRI or NI), TCAs that act on various distinct targets such as serotonergic, adrenergic and cholinergic systems and fast voltage-gated sodium channels, and SNRIs. **(B)** Examples of emerging potential targets and therapeutics for neuropathic pain. 5-HT: 5-hydroxytryptamine, CB1/2: cannabinoid receptor type 1/2, Ca_V_: voltage-gated calcium channel, EGF: epidermal growth factor, EGFR: epidermal growth factor receptor, EREG: epiregulin, IL: interleukin, IL-6R: interleukin-6 receptor, IL-1R: interleukin-1 receptor, mAChR: muscarinic acetylcholine receptor, MOR: mu-opioid receptor, Na_V_ voltage-gated sodium channel, NGF: nerve growth factor, NI: norepinephrine reuptake inhibitor, SNRI: selective serotonin reuptake inhibitor, TCA: tricyclic antidepressant, TNF: tumor necrosis factor, TNFR: tumor necrosis factor receptor, TrkA: tropomyosin receptor kinase A, TRPV1: transient receptor potential cation channel subfamily V member 1.

Current pharmacological agents used for neuropathic pain span a wide breadth of drug classes. As shown in Figure 1A and extensively reviewed elsewhere, these include gabapentinoids, antidepressants, namely tricyclic antidepressants (TCAs) and selective serotonin and norepinephrine reuptake inhibitors (SNRIs), topical agents (lidocaine and capsaicin), and opioids (Alper and Lewis, 2002; Rauck et al., 2009; Kremer et al., 2016; Chincholkar, 2018; Coderre, 2018; Schembri, 2019; Windsor et al., 2019; Ardeleanu et al., 2020; Kapustin et al., 2020; Gress et al., 2020; Chalil et al., 2021). Use of these and other analgesic medication classes is often limited by inadequate efficacy or side effects, necessitating the ongoing identification of novel drug targets. To that end, a variety of emerging potential therapeutic agents for neuropathic pain are being repositioned or developed for clinical trial. These include monoclonal antibodies against inflammatory mediators, cannabinoids, and inhibitors of G protein-coupled receptors and N-methyl-D-aspartate receptors (Figure 1B) (Karppinen et al., 2003; Stahel et al., 2016; Jung et al., 2017; Wang et al., 2017; Aiyer et al., 2018; Mapplebeck et al., 2018; Kreutzwiser and Tawfic, 2019; Alkislar et al., 2020; Haleem and Wright, 2020; Kushnarev et al., 2020; Maayah et al., 2020; Yu et al., 2020; Zhang W. et al., 2020; Matarazzo et al., 2021).

Here, we focus on EGFR as an important mediator of neuropathic pain and an emerging therapeutic target. The first clinical evidence for the involvement of EGFR in neuropathic pain was observed in cancer-induced neuropathic pain and later expanded to non-cancer patients (Kersten and Cameron, 2012; Kersten et al., 2013). In what follows, we present the clinical trial and genetic association data linking EGFR to pathological pain states, with an emphasis on human neuropathic pain.

### Clinical Studies

Clinical evidence provided through case reports and clinical trials suggest that therapeutics targeting EGFR may alleviate pain in both cancer patients and non-cancer patients afflicted with neuropathic pain. Phase III clinical trials have reported that a significant proportion of patients with non-small cell lung cancer (NSCLC) experienced pain relief following treatment with erlotinib (Bezjak et al., 2006; Cappuzzo et al., 2010) in addition to significant improvements in physical functioning and quality of life (Bezjak et al., 2006). A phase III clinical trial of afatinib, a tyrosine kinase inhibitor of ErbB family proteins (EGFR, HER2, HER3 and HER4), also reported that a significant proportion of patients with advanced NSCLC experienced pain relief (Hirsh et al., 2013). However, in these studies, it remains unclear as to whether pain relief was attributable to the effects of EGFR therapy on the tumor or a direct effect of EGFR therapy on neuropathic pain signaling.

A case study by Kersten and colleagues reported that a patient with rectal cancer experienced pain relief with cetuximab treatment despite tumor progression (Kersten and Cameron, 2012). Notably, administration of 20% the patient’s normal cetuximab dose did not relieve pain, suggesting cetuximab-induced pain relief was not due to placebo effect. A follow-up case series reported that four of five cancer and non-cancer patients with neuropathic pain, two male and two female patients, experienced a self-reported reduction in pain from 9 to 1 on a 10-point scale shortly after intravenous administration of cetuximab or panitumumab, two antibody therapies directed against EGFR (Kersten et al., 2013). Another study by Kersten and colleagues reported that cetuximab treatment provided pain relief for eighteen out of twenty patients afflicted with neuropathic pain, including cancer and non-cancer patients of both sexes (Kersten et al., 2015). In a randomized control trial, male and female neuropathic pain patients experienced the greatest average pain reduction following open-label or blinded cetuximab treatment, and the lowest average pain reduction with the blinded placebo (Kersten et al., 2019). Importantly, unlike many current treatment options for neuropathic pain, the side effects observed in patients treated with various therapeutics that target EGFR, including cetuximab and panitumumab (monoclonal antibody therapeutics) and/or erlotinib and gefitinib (tyrosine kinase inhibitors, TKI)**,** were generally mild to moderate (Kersten et al., 2015; Kersten et al., 2019). Together, these data suggest EGFR as a potential therapeutic target for patients afflicted with neuropathic pain that in the treatment of cancer-related neuropathic pain may be at least partly distinct from the effect of EGFR therapies on the tumor itself. Understanding the mechanism by which the EGFR signaling pathway contributes to neuropathic pain is an important research priority that may lead to improved outcomes for patients with neuropathic pain.

### Genetic Links

There is a growing body of genetic studies that are identifying important pathways in acute and chronic pain. One such study is the Orofacial Pain Perspective Evaluation Risk Assessment (OPPERA), which examined the environmental, biosocial, and genetic factors that may lead to painful temporomandibular disorders (Fillingim et al., 2011; Maixner et al., 2011; Slade et al., 2013). Subsequent genetic analyses of the OPPERA study cohorts revealed single nucleotide polymorphisms (SNPs) of EGFR and one of its cognate ligands, epiregulin (EREG), that may be associated with the development of chronic pain in temporomandibular disorders (Martin et al., 2017; Verma et al., 2020). Specifically, EREG and EGFR SNPs have a high association with the development of temporomandibular disorders in European females (Martin et al., 2017), with the variants of EREG SNPs decreasing circulating EREG mRNA (Verma et al., 2020). This decrease was found to be associated with protection against chronic pain but paradoxically increases the risk of systemic hypersensitivity in acute pain (Verma et al., 2020). This clinical observation is consistent with animal studies in which antibody inhibition of circulating EREG demonstrates similarly opposing effects in acute and chronic pain (Verma et al., 2020). Collectively, these studies suggest that EGFR and EREG may have roles in the development of chronic pain but may also protect against sensitization in acute pain. However, due to the limitations of these cohorts, including small sample sizes and limited evaluation of sex as an important biological variable, additional studies are required. Future work may include broader and diverse samples for genetic analyses and sex-specific *in vivo* studies will allow validation of the possible link between EREG and chronic pain across different patient populations.

Transcriptional profiling of macrophages from synovial fluids of patients suffering from rheumatoid arthritis (RA) and pancreatic cancer, has further implicated potential roles of the EGFR signaling pathway in pain (Wangzhou et al., 2021). This was revealed by an interactome map of human DRG RNA-seq and macrophages derived from patient synovial tissue (single-cell RNA-seq). Three of the transcripts enriched in the RA macrophages include heparin-binding EGF-like growth factor (HB-EGF), EREG and decorin, a TGF-β derived proteoglycan binding peptide, that when released by the macrophage have the potential to activate EGFR in the DRG. A similar analysis using bulk RNA-seq of pancreatic cancer patients and non-cancer patients revealed that of the 41 ligands that are upregulated in cancerous tissues, four activate or modulate EGFR, including the EGFR ligand TGF-α, and modulators CEACAM1, FGF1, and TFF1 (Wangzhou et al., 2021). While these associations do not directly entail causality, mechanistic *in vivo* evidence is limited to the use of animal models. Thus, these data highlight potential interactions in human tissues–specifically, between EGFR within the DRG and ligands associated with pain-associated diseases and underscore the importance of investigating the role of EGFR in chronic pain.

## Epidermal Growth Factor Receptor

EGFR has central roles in development and in the maintenance of homeostasis in adult tissues, including the central and peripheral nervous systems, where it exerts neurotrophic actions (Aguirre et al., 2007; Chong et al., 2008; Garcez et al., 2009; Hu et al., 2010; Sinor-Anderson and Lillien, 2011). Ligand binding and activation of EGFR occurs within the plasma membrane. Structurally, EGFR contains an extracellular region, the ligand-binding domain, transmembrane region, and intracellular region containing an intrinsic tyrosine kinase domain and a c-terminal tail that harbors many tyrosine residues that become phosphorylated upon ligand binding (Lemmon and Schlessinger, 2010). EGFR is one of four members of the ErbB family of RTKs, the others being HER2, HER3 and HER4. These family members bind different ligands (or no ligand in the case of HER2), and form various homo- or heterodimers [for review see Lemmon et al. (2014)]. EGFR may bind various ligands that are characterized into two groups based on their binding affinity: high-affinity ligands include EGF, transforming growth factor alpha (TGF-α), betacellulin and (HB-EGF); and low affinity ligands include amphiregulin, epiregulin (EREG), and epigen (Freed et al., 2017). The binding of high- or low-affinity ligands not only leads to the formation of distinct structural dimers, but generates transient or sustained signalling, respectively, and thus different cellular physiological outcomes (Freed et al., 2017).

**FIGURE 2:**
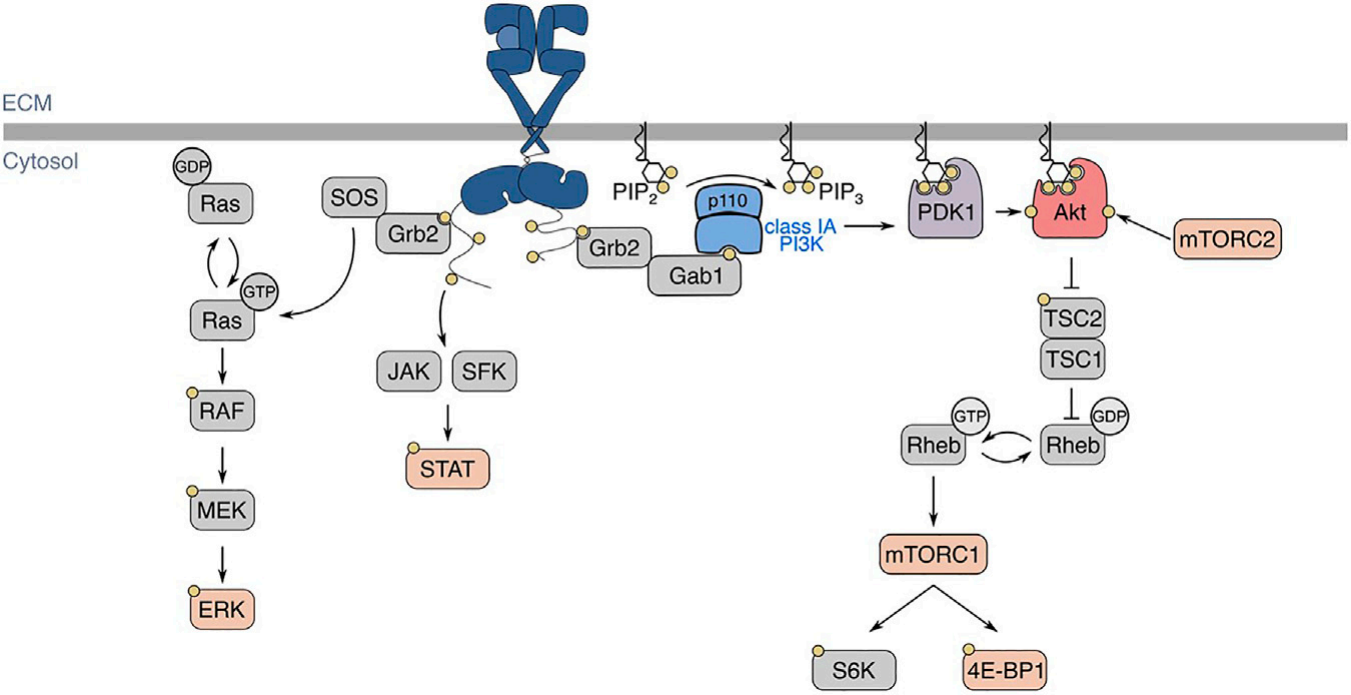
EGFR structure, ligand-binding, and signaling pathways. In the basal state, EGFR exists in an autoinhibited conformation. Ligand-binding allows for the formation of homo- or heterodimers, which activates EGFR intrinsic tyrosine kinase domains and ensues signal transduction, shown here as an EGFR homodimer with one ligand bound. Various signalling pathways are initiated by the active EGFR, such as Ras-Erk, JAK/SFK-STAT, and PI3K-Akt. Regarding EGFR-PI3K-Akt signalling, EGFR autophosphorylation at Y1068 allows for the activation of class IA PI3K. The active PI3K subsequently phosphorylates PI(4,5)P_2_ (PIP_2_) into PI(3,4,5)P_3_, which recruits kinases PDK1 and Akt to the plasma membrane. There, Akt is phosphorylated by PDK1 and mTORC2 at T308 and S473, respectively. EGFR: epidermal growth factor receptor, JAK: Janus kinase, SFK: Src-family kinase, STAT: signal transducer and activator of transcription, PI3K: phosphoinositide 3-kinase, PDK1: phosphoinositide-dependent kinase 1, mTORC: mechanistic target of rapamycin complex, TSC: tuberous sclerosis complex, SOS: son of sevenless, Grb2: growth factor receptor-bound protein 2, Gab1: Grb2-associated binding protein 1.

Depending on the cellular expression of HER2, HER3, and HER4, ligand-binding to EGFR induces receptor homo- or heterodimerization, and the activation of intrinsic tyrosine kinase domains that leads to phosphorylation of the receptor’s c-terminal cytosolic tail (Lemmon et al., 2014). These phosphotyrosine residues are part of motifs that allow binding of various SH2- and PTB-domain containing proteins, leading to the subsequent activation of various intracellular signaling pathways, such as phosphatidylinositol-3-kinase (PI3K)-Akt, Ras-Erk, and signal transducer and activator of transcription (STAT) proteins (Han and Lo, 2012; Sugiyama et al., 2019) (Figure 2). Following ligand binding at the cell surface, EGFR undergoes internalization and intracellular vesicle traffic, a phenomenon that regulates EGFR signaling in multiple ways (Vieira et al., 1996; Sigismund et al., 2008; Garay et al., 2015; Reis et al., 2015; Villaseñor et al., 2015; Pinilla-Macua et al., 2016; Chen et al., 2017; Leyton-Puig et al., 2017; Pascolutti et al., 2019; Thapa et al., 2020). The complexity of these signaling pathways is underscored by the compartmentalization of signalling intermediates of various isoforms, and membrane traffic of these proteins and the receptor–all of which have important roles in contributing to signal fidelity and specificity (Sugiyama et al., 2019).

### Epidermal Growth Factor Receptor in Animal Models of Pain

Animal models of pain are important and necessary to understand fundamental mechanisms of disease and to identify new therapeutic targets (Mogil et al., 2010). Various animal models of pain have been designed to mimic distinct clinical pathologies (Gregory et al., 2013). For example, models of neuropathic pain include spared nerve injury, in which the sciatic nerve undergoes partial injury (Cichon et al., 2018), spinal nerve ligation (Chung et al., 2004), and paclitaxel-induced neuropathy (Griffiths et al., 2018). Pain perception varies among individuals and an analogous heterogeneous response to nociceptive stimuli can be observed in rodents. As pain cannot be measured directly in animal models, methods that quantify nociceptive behaviours have been developed (reviewed by (Deuis et al., 2017) as a surrogate outcome.

EGFR has been shown to mediate hypersensitivity in various animal models of pain. Systemic administration of various EGFR inhibitors (AG-1478, gefitinib, lapatinib) reverses complete Freund’s adjuvant (CFA)-, spared nerve injury (SNI)- and chronic constriction injury (CCI)-induced mechanical allodynia, as well as carrageenan-induced thermal hypersensitivity (Martin et al., 2017). Systemic administration of these inhibitors also reduces nociceptive behaviours in the late phase of the formalin test (Martin et al., 2017). However, a recent report has shown that gefitinib administration does not block spinal nerve ligation (SNL)-induced mechanical allodynia, though the authors suggest that the discrepancy between this and previous findings may be due to differences in SNI and SNL models of neuropathic pain (Puig et al., 2020). In the SNL model, the L5 and L6 spinal nerves are ligated whereas in the SNI model, the tibial and common peroneal nerves are ligated and sectioned 2–3 mm from the ligation, leaving the sural nerve intact (Challa, 2015). The methodology of and challenges associated with each of these models have been described previously [reviewed by (Challa, 2015)]. Intrathecal delivery of gefitinib or EGFR siRNA reverses chronic constriction of the DRG (CCD)-induced mechanical, heat, and cold hypersensitivity (Wang et al., 2019). Inhibition of EGFR with cetuximab attenuates nociceptive behaviors in a mouse model of oral cancer pain (Scheff et al., 2020). Given these data, it appears that EGFR may have roles in driving hypersensitivity across multiple pain models, though this may vary with context.

### Epidermal Growth Factor Receptor Is Expressed Within the Dorsal Root Ganglion and Spinal Dorsal Horn

The dorsal horn, dorsal root ganglia, and trigeminal ganglia are important areas in pain modulation (Ji et al., 2016; Esposito et al., 2019), and multiple studies have detected EGFR expression within primary afferents and immune cells within these areas (Werner et al., 1988; Puig et al., 2020). Immunohistochemistry (IHC) staining for EGFR in primary afferents suggest a heterogeneous expression pattern within afferent nerve fibers of various diameters in human and rat DRG (Werner et al., 1988; Birecree et al., 1991; Huerta et al., 1996; Martin et al., 2017). Recent studies have demonstrated that EGFR may be broadly expressed in many different sensory fiber types as indicated by cellular colocalization of EGFR with NF200, CGRP, and IB4 in the rat DRG and spinal cord (Wang et al., 2019; Puig et al., 2020). These data imply that EGFR is expressed in myelinated, unmyelinated peptidergic, and unmyelinated non-peptidergic fibers, respectively. However, within the spinal cord, EGFR does not colocalize with neuron-specific nuclear protein (NeuN), suggesting second-order neurons may not express EGFR (Puig et al., 2020). IHC staining of other ErbB family proteins (HER2, HER3 and HER4) demonstrates that these receptors are also differentially expressed within the DRG and dorsal horn (Pearson and Carroll, 2004). Collectively, these studies establish EGFR expression in many different afferent nerve fibres.

EGFR expression has also been detected in non-neuronal cell types, namely immune and glial cells, within the DRG and dorsal horn. Microglia, the resident macrophages of the CNS, may have key roles in the establishment and maintenance of neuropathic pain (Tsuda, 2016). Cellular colocalization of EGFR with OX42 (Puig et al., 2020) and western blotting of isolated (spinal) microglia suggest microglia within the rat spinal cord express EGFR (Qu et al., 2012). Satellite glial cells of the DRG may have roles in neuropathic pain by releasing soluble pain mediators, such as growth factors and cytokines (Wei et al., 2019). IHC of human DRGs suggests satellite and interstitial Schwann cells express EGFR (Werner et al., 1988), and colocalization of EGFR with glutamate synthase suggests satellite cells express EGFR within the rat DRG (Wang et al., 2019). Though some reports have shown that astrocyte expression of EGFR is downregulated within the postnatal rat brain (Scholze et al., 2014), other studies have shown that EGFR is expressed within astrocytes and other cell types, possibly macrophages, within the gray matter of the female rat spinal cord (Erschbamer et al., 2007; Li et al., 2014). Collectively, these studies provide evidence that EGFR is expressed both in sensory neurons as well as in immune and supportive cells relevant to pain, providing an anatomic and cellular substrate for several possible mechanisms of action of EGFR in neuropathic pain.

### Epidermal Growth Factor Receptor is Upregulated Within the Dorsal Root Ganglion in Various Pain Models

EGFR mRNA increases in the ipsilateral DRG of male rats following SNL or CCD (Wang et al., 2019) as well as spinal cord injury (SCI) (Erschbamer et al., 2007; Li et al., 2014). This increase in EGFR mRNA was also observed following SCI in a subpopulation of sensory neurons within the DRG (Erschbamer et al., 2007). Contact of vertebral pulposus (NP) tissue with peripheral nerves has been used to emulate the contribution of inflammatory mediators secreted by NP tissue to pain associated with radiculopathy (Takebayashi et al., 2001; Cuellar et al., 2005; Egeland et al., 2013). Contact of NP tissue with exposed dorsal nerve roots leads to increased HER3, but not EGFR, HER2 or HER4 mRNA within the DRG (Kongstorp et al., 2019), suggesting other ErbB proteins may have roles in mediating pain. Given that HER3 and EGFR may form heterodimers, how the regulated expression of EGFR and other HER family members observed following nerve lesions may alter nociception and contribute to neuropathic pain warrants further investigation.

EGFR phosphorylation at Y1068 leads to PI3K-Akt signaling, a phenomenon that may be increased within the DRG in various models of pain, including CFA and SNI (Martin et al., 2017). EGFR is also phosphorylated at Y1068 within the ipsilateral DRG following CCD, but is not activated within the contralateral DRG or within the spinal cord (Wang et al., 2019). Thus, given the evidence published to date, EGFR phosphorylation (Y1068) may be upregulated within the DRG in inflammatory and neuropathic pain models.

Since EGFR may be activated directly by ligand-binding or indirectly through transactivation by other receptors, we next discuss EGFR ligands and their implicated roles in pain.

### Direct Actions of Epidermal Growth Factor Receptor by Ligand-Binding

A key regulatory mechanism of RTK signaling involves controlling the availability of active-form ligands to the receptor. Many RTK ligands are initially synthesized as transmembrane proteins (Adrain and Freeman, 2014) and in the case of EGFR, pro-ligands are made soluble by the membrane-bound metalloprotease, ADAM-17, which proteolytically processes more than 80 substrates including cytokines, adhesion molecules and cognate ligand precursors of EGFR (Quarta et al., 2019). Multiple ADAM-17 substrates are involved in pain hypersensitivity and inflammation, and as such, recent studies have sought to elucidate the role of ADAM-17 in nociception and pain sensitization. Scheff and colleagues demonstrated that the supernatant of some oral cancer cell lines is sufficient to induce nociceptive behavior in mice, which was attenuated by EGFR inhibition, and that this supernatant contained higher levels of ADAM17 than non-nociceptive cell lines. Importantly, pathway analysis revealed enhanced PI3K-Akt and mTORC1 signaling upon treatment with nociceptive oral cancer cell supernatant (Scheff et al., 2020). Together, these data suggest a model in which cleaved targets of ADAM-17 may induce hyperalgesia through the EGFR signaling axis. However, establishing a causal link between elevated ADAM17, EGFR ligands and nociception and pain sensitization requires additional research, as some ADAM substrates may transactivate EGFR, including IL-6, which in ovarian cancer, may exhibit crosstalk with EGFR in both its membrane-bound and soluble state (Colomiere et al., 2009). However, consistent with these studies, other evidence further suggests that ADAM-17 may have roles in neuropathic pain, as levels of ADAM17 mRNA increase in the ipsilateral dorsal horn following partial sciatic nerve ligation of mice (Brifault et al., 2019). Additionally, hypomorphic ADAM-17 (ADAM-17^ex/ex^) mice have elevated mechanical thresholds and impaired sensitivity to heat and cold and the DRG of ADAM-17^ex/ex^ mice contain fewer β-4 positive neurons, which are canonically responsive to thermal nociceptive stimuli (Quarta et al., 2019).

Following binding of active-form ligand, the activation of EGFR allows for various signaling pathways to take place that may drive hypersensitivity. There is a wealth of information about the specific roles and actions of distinct EGFR and ErbB ligands in a variety of physiological and pathophysiological settings [reviewed by Citri and Yarden (2006), Ho et al. (2017)]. Importantly, some EGFR ligands of both high and low affinity have been implicated in mediating pain associated with various diseases such as temporomandibular disorder (Martin et al., 2017), vertebral disc herniation and degeneration and radiculopathy (Huang et al., 2017; Kongstorp et al., 2019), rheumatoid arthritis and cancer (Wangzhou et al., 2021) (summarized in Table 1).

**TABLE 1:** Summary of EGFR ligands and their implicated actions in pain.

Ligand	Receptor(s)	Evaluated nocifensive stimuli	Mechanistic findings	Implicated human pain conditions	References
High affinity ligands					
EGF	EGFR	Mechanical	GRK2 phosphorylation; opioid receptor downregulation	Opioid tolerance	Chen et al. (2008), Puig et al. (2020)
BTC	EGFR, HER4	—	—	—	—
HB-EGF	EGFR, HER4	Mechanical	↑ DRG neuron intracellular Ca^2+^	RA	Wangzhou et al. (2021)
TGFα	EGFR	—	—	Pancreatic cancer	Wangzhou et al. (2021)
Low affinity ligands					
AREG	EGFR	—	—	—	—
EREG	EGFR, HER4	Heat; capsaicin; mechanical; formalin	PI3K-Akt-mTORC1 signaling activation; ↑ spontaneous C fibre firing; ↑ capsaicin-evoked DRG neuron intracellular Ca^2+^	RA; radiculopathy; peripheral nerve injury	Huang et al. (2017), Martin et al. (2017), Kongstorp et al. (2019), Wangzhou et al. (2021)
EPGN	EGFR	—	—	—	—

Experimental evidence for and suggested roles of EGFR ligands in pain. Evaluated nocifensive stimuli, mechanistic findings and human conditions in which ligands have been implicated to contribute to pain are indicated. EGF: epidermal growth factor, BTC: betacellulin, HB-EGF: heparin-binding epidermal growth factor-like growth factor, TGFα: transforming growth factor alpha, AREG: amphiregulin, EREG: epiregulin, EPGN: epigen. PGE2: prostaglandin E2. i. t.: intrathecal, DRG: dorsal root ganglion, RA: rheumatoid arthritis, SNI: spared nerve injury, CFA: complete Freund’s adjuvant. — indicates no available data.

Though some EGFR ligands have been specifically investigated, the contribution of others in pain signaling can only be inferred and require experimental confirmation. Thus, we focus here on EREG and EGF which have been investigated most thoroughly in this context.

#### Epidermal Growth Factor Receptor Ligands and Their Roles in Pain

EREG, but not other EGFR ligands (EGF, amphiregulin, betacellulin, TGF-α), enhances formalin-induced nocifensive behaviors in mice with EREG administration alone being sufficient to induce heat and mechanical hypersensitivity (Martin et al., 2017). For comparison, EREG-enhanced formalin-induced nocifensive behaviours are comparable to those produced by an established pain modulator, NGF. Importantly, inhibition of the NGF receptor, TrkA, does not block EREG-induced effects, suggesting the mechanism by which EREG promotes pain occurs independently of NGF receptor activity (Martin et al., 2017).

Whether EREG is the only EGFR ligand capable of inducing mechanical hypersensitivity has been addressed recently: EGF administration alone is sufficient to induce mechanical but not thermal hypersensitivity (Puig et al., 2020). However, this effect takes repeated administration over several days to take place, while EREG-induced hypersensitivity occurs within an hour (Puig et al., 2020). Intraplantar administration of HB-EGF is sufficient to induce mechanical sensitivity in male and female mice (Wangzhou et al., 2021), which peaks 1 h after HB-EGF administration after which animals recover to an extent over the course of days.

Whether differences in ligand affinity may contribute to these potential differences remains to be determined. TGF-α may have potential roles in pancreatic cancer pain (Wangzhou et al., 2021)**.** However, whether TGF-α administration alone is sufficient to induce hypersensitivity remains to be investigated. Thus, whether other EGFR ligands may participate in sensitization of other contexts is as of yet unclear. Different EGFR ligands may distinctly contribute to neuropathic pain by different patterns of expression and secretion, or by distinct regulation of the magnitude or duration of EGFR signaling following ligand binding. Collectively, this diversity of EGFR ligand regulation may contribute to context-dependent action of each EGFR ligand in promoting pain sensitization, though further mechanistic studies are required to fully delineate whether this is indeed the case.

Despite the complexity of EGFR ligand signaling, there has been substantial evidence to support a key role for EREG in pain signaling. Following lumbar vertebral disc herniation, EREG is released from intervertebral disks by the NP and annulus fibrosus in patients (Huang et al., 2017), or by NP cells in female rats (Kongstorp et al., 2019). Application of NP-derived EREG onto spinal dorsal nerve roots reduces evoked C-fiber responses but increases their spontaneous activity (Kongstorp et al., 2019), and thus, EREG may act as to mediate neuropathic pain through peripheral mechanisms.

As described above, recent evidence has demonstrated that inhibition of EREG reverses or enhances hypersensitivity in chronic or acute pain models, respectively (Verma et al., 2020). This suggests that EREG mitigates pain during the early stages of its development but eventually contributes to the establishment of chronic pain (Verma et al., 2020). It remains to be elucidated as to whether EREG has direct roles in the establishment of chronic pain or in the maintenance of the chronic pain state.

As both EGFR and its ligands can be expressed in various immune cells relevant to pain and nociception, EREG may have roles in inflammation-driven pain. Clinical data shows increased levels of EREG in leukocytes of patients with temporomandibular disorders (Martin et al., 2017) and RA (Wangzhou et al., 2021). Levels of circulating EREG increase following CFA and SNI (Martin et al., 2017) in rats. Thus, we next discuss EREG and its roles in inflammation in the context of pain.

#### Epiregulin May Modulate Pain by Altering Inflammation

The inflammatory responses involved in both injury and various diseases drive the development and maintenance of neuropathic pain by promoting mechanical allodynia, neuronal hypersensitivity, and central sensitization (Baral et al., 2019). EGFR and its cognate ligands have various roles in inflammation, which may also contribute to neuropathic pain pathogenesis. Following their activation, various immune cells secrete EGFR ligands (Burgel and Nadel, 2008). Macrophages exhibit enhanced levels of EREG mRNA following LPS stimulation (Oshima et al., 2011). Primary microglia express membrane-bound EGF (Coniglio et al., 2012), and in mice, EGF expression is enhanced 4 days following spinal cord contusion injury (Greenhalgh et al., 2018).

EREG contributes to inflammation, in part, by modulating the expression of cytokines and growth factors in inflammatory diseases (Murakami et al., 2013; Harada et al., 2015). The expression of pro-inflammatory cytokines in the spinal cord, DRG, injured nerves or skin are associated with pain behaviors and the development of abnormal spontaneous activity from compressed, injured and inflamed DRG neurons (Zhang and An, 2007). Reports first demonstrated that EREG may act synergistically with cytokines to propagate the inflammatory response as the ligand itself is expressed following *in vitro* stimulation with interleukin (IL)-6 and IL-17 (Murakami et al., 2013). EREG subsequently further stimulates an increase in EREG mRNA expression as well as that of IL-6, and enhances NFκB signaling through PI3Kα, IKKα or IKKγ (Murakami et al., 2013). A follow-up study demonstrated that the serum of individuals with RA have elevated levels of several growth factors including EREG, an observation also made in rodent models of RA (Harada et al., 2015). Importantly, the study reports that only EREG levels increase during the early phase of inflammation, while other growth factors were present in the joints at later phases of inflammation (Harada et al., 2015). Additionally, *in vitro* analysis of joint synovial fluid in mice with cytokine-induced RA demonstrates that the neutralization of EREG decreases the expression of growth factors and thereby downregulates further EREG expression (Harada et al., 2015). This collectively suggests that EREG may have a key role in the development of inflammation and pain, however further studies evaluating the functional role of EREG in inflammatory processes are required.

#### Epiregulin-Epidermal Growth Factor Receptor Signaling Mechanisms That May Contribute to Hyperalgesia and Allodynia

Intrathecal administration of EREG triggers signaling cascades within the DRG that promote pain sensitization (Martin et al., 2017). Inhibition of PI3K or mTORC1, but not MEK1/2, blocks EREG-enhanced pain behaviors following formalin administration, suggesting EREG-enhanced hypersensitivity occurs by a PI3K- and mTORC1-dependent, MEK-independent mechanism (Martin et al., 2017). Here, we describe the signaling cascades activated by EGFR and how the resultant activation of the intermediate proteins PI3K, mTORC1 and MAPK drive peripheral and central sensitization.

##### Phosphatidylinositol 3-Kinase

PI3Ks are lipid kinases that catalyze the phosphorylation of the 3-position of phosphoinositol. There are three classes of PI3Ks, each generally responsible for generating a different phosphoinositide (phosphorylated derivative of phosphatidylinositol) (Jean and Kiger, 2014). The various phosphoinositides generated by PI3K act upstream of many signaling pathways. While PI3Ks are canonically known to drive cellular growth, PI3K have also been widely shown to mediate pain in various contexts. Inhibition of PI3K using wortmannin (Xu et al., 2011; Guo et al., 2017), which may inhibit all classes of PI3K (McNamara and Degterev, 2011), or LY24002 (LY) (Liu et al., 2018), which inhibits class I and III PI3K (Gharbi et al., 2007), perturbs CCI-induced mechanical and thermal hyperalgesia in male rats. In male rodents, inhibition of PI3K using wortmannin reduces carrageenan-induced thermal and mechanical hypersensitivity, as well as late-phase formalin-induced pain behaviors (Xu et al., 2011).

PI3Ks have been shown to contribute to both the establishment and maintenance of neuropathic pain, of which class I PI3Ks have been implicated to have significant roles. However, there remains limited understanding of the importance of the different class I PI3K isoforms in pain. Specific class I PI3K isoforms, which catalyze the phosphorylation of phosphatidylinositol-4,5-bisphosphate (PI(4,5)P_2_) into phosphatidylinositol-3,4,5-trisphosphate (PIP_3_), are differentially expressed within the nervous system. The differential distribution of class I PI3K isoforms (α, β, γ, δ) within various neuron types, supportive cells and immune cells within the DRG and spinal dorsal horn has been implicated in mechanisms of pain (Leinders et al., 2014). After 4 h of intraplantar carrageenan administration, mRNA levels of PI3K α and PI3Kβ or PI3Kγ and PI3Kδ significantly increase within the DRG or spinal cord, respectively. Intrathecal pre-treatment with a PI3Kβ inhibitor blocks carrageenan-induced mechanical allodynia, while inhibitors specific to other PI3K isoforms are not capable of blocking carrageenan-induced mechanical allodynia (Leinders et al., 2014). In contrast, pre-treatment with a PI3Kγ-specific inhibitor but not inhibitors of other PI3K isoforms blocks intraplantar carrageenan-induced mechanical allodynia. Additionally, protein levels of some class I PI3K catalytic and regulatory subunits increase following CCI (Liu et al., 2018). Collectively, these data suggest that the differential distribution of class I PI3K isoforms have distinct roles in nociception. However, the roles of class I PI3K isoforms in different models of pain and in response to other nociceptive stimuli remain to be elucidated. Thus, while there is evidence of upregulation of, and a functional role for class I PI3Ks in pain, further investigation into their precise contributions to pain is warranted.

**FIGURE 3:**
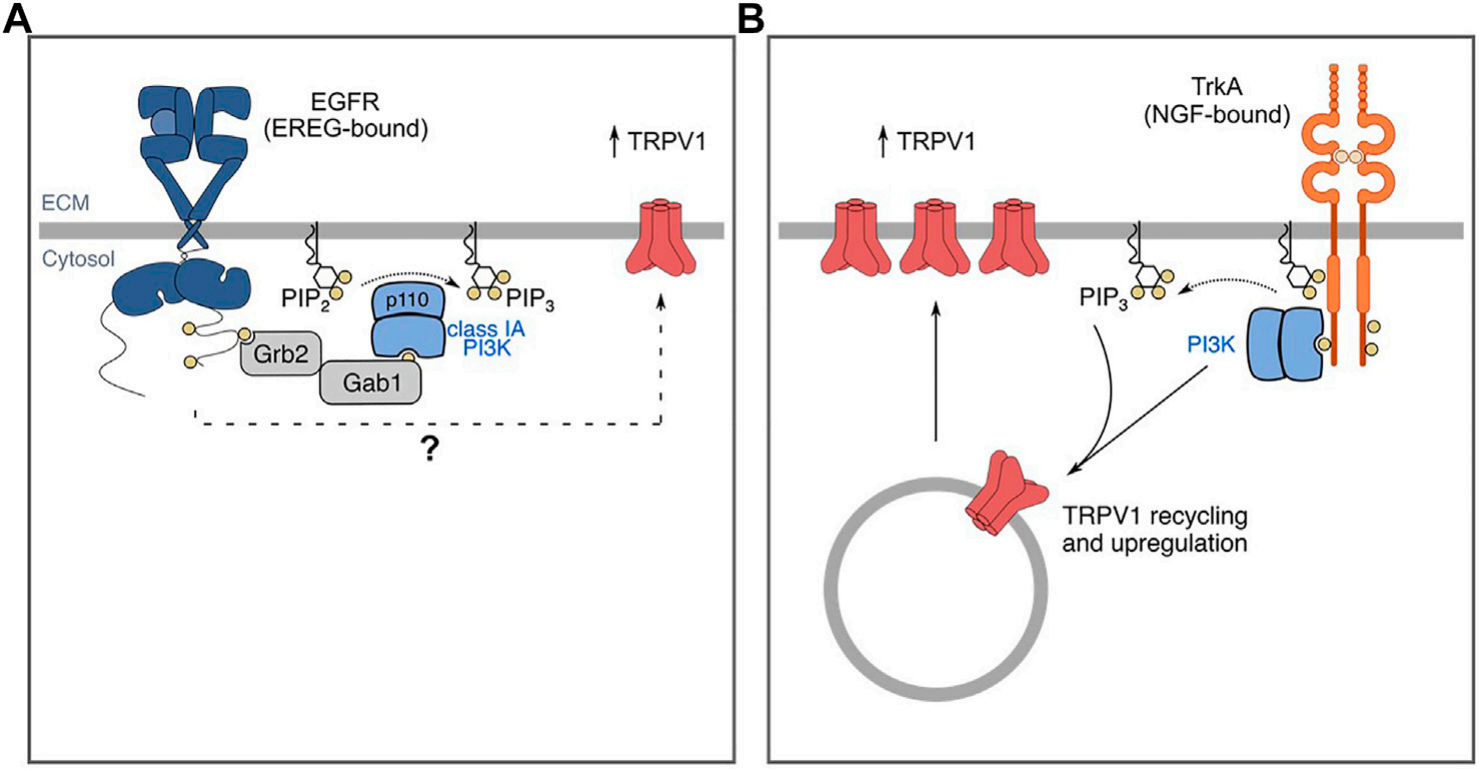
EGF/EREG-EGFR and NGF-TrkA signaling mechanisms that contribute to pain/sensitization. **(A)** EREG-mediated sensitivity to formalin and capsaicin involves TRPV1. **(B)** Similarly, NGF mediates sensitivity to capsaicin and formalin. NGF-TrkA signalling mediates traffic of TRPV1 to the plasma membrane through a PI3K-dependent mechanism. The mechanisms by which NGF mediates sensitivity through these signaling intermediates is more well-characterized and thus, the mechanisms by which EREG-EGFR signaling promotes sensitivity warrants further investigation. The mechanisms by which other EGFR ligands mediate sensitivity are incompletely understood. Additionally, whether and how EREG-EGFR and EGF-EGFR signaling may promote sensitivity to distinct stimuli in various animal models remain to be elucidated.

There is also some evidence that PI3K contributes specifically to EGFR-dependent pain (Figure 3A). PI3K inhibition by wortmannin attenuates EREG-enhanced formalin-induced nociceptive behaviors, suggesting EREG-mediated hyperalgesia occurs through a PI3K-dependent mechanism (Martin et al., 2017). However, it should be noted that PI3K is a common signaling intermediate for many receptors implicated in pain and as such, PI3K may be required by other growth factor receptors (namely RTKs) that mediate pain, such as FGFR (Zhang M. et al., 2020). Consistent with this, NGF-TrkA-mediated pain occurs through a PI3K-dependent mechanism (Bonnington and McNaughton, 2003). Hence, further mechanistic study of how PI3K may specifically mediate pain signaling downstream of EGFR in neurons and/or relevant immune cells in the context of pain will be very informative.

One protein that is affected by EREG and/or PI3K activation is the transient receptor potential cation channel subfamily V member 1 (TRPV1). TRPV1 has been widely studied for its roles in thermal hyperalgesia and responds to polymodal stimuli including noxious heat and vanilloid substances such as capsaicin (Jardín et al., 2017). Administration of EREG alone is sufficient to induce thermal hyperalgesia (Martin et al., 2017). In addition, EREG enhances capsaicin-induced nocifensive behaviors. Administration of the TRPV1 antagonist AMG 9810 blocks EREG-induced hyperalgesia in the late phase of the formalin test (Martin et al., 2017). These data suggest that one of the mechanisms by which EREG may promote sensitivity involves TRPV1. However, whether EREG enhances TRPV1 activity through mechanisms such as transcriptional regulation, or by enhancing cell surface abundance, remain to be determined (Figure 3A). Similarly, NGF administration has also been reported to promote sensitization in part by inducing TRPV1 upregulation through a PI3K-dependent mechanism (Figure 3B) (Stein et al., 2006; Zhu and Oxford, 2007a; Stratiievska et al., 2018).

The mechanism by which NGF mediates TRPV1 upregulation has been studied in greater detail than that of EREG. In F-11 cells, a cellular model of undifferentiated sensory neurons, NGF-stimulation leads to increased cell surface TRPV1 (Stein et al., 2006; Stratiievska et al., 2018), and inhibition of PI3K with wortmannin blocks this effect (Stratiievska et al., 2018). NGF stimulation enhances plasma membrane TRPV1 with constant cytosolic TRPV1 (Pinho-Ribeiro et al., 2017). It is unclear as to whether TRPV1 may be transcriptionally upregulated or whether this enhanced traffic to the cell surface occurs with constant total TRPV1 expression, the latter of which would indicate NGF mediates TRPV1 traffic to the cell surface. In addition, patch-clamp recordings of mouse DRG neurons suggests that NGF stimulation leads to an increase in maximum current, but not the EC50 in response to capsaicin through enhanced cell-surface TRPV1 (Stein et al., 2006). Some evidence suggests that the regulation of TRPV1 and PI3K may be reciprocal.

Class I PI3K activation canonically leads to the phosphorylation and activation of Akt and although some studies suggest roles for Akt in hypersensitivity following formalin administration (Xu et al., 2011), others suggest Akt may not have a significant role as Akt phosphorylation may decrease in the late phase of the formalin test, but this observation was not statistically significant (Pezet et al., 2008). Thus, whether and how Akt may be involved in sensitization by EREG or other EGFR ligands remains poorly understood.

In F-11 cells, in addition to PI3K signaling leading to enhanced cell surface TRPV1, ectopic TRPV1 expression enhances NGF-stimulated PI3K-dependent Akt phosphorylation at T308 and S473, both of which are required for full activation of Akt (Stratiievska et al., 2018). Furthermore, NGF-enhanced capsaicin-induced nocifensive behaviors are greatly reduced in PI3K-deficient mice (p85α-null), suggesting that class I PI3K is important in driving sensitization (Zhu and Oxford, 2007a). It remains to be elucidated as to whether EREG-induced sensitization through TRPV1 requires specific PI3K subunit isoforms, and whether EGFR augments TRPV1 cell surface expression. Like EREG, NGF may be produced by immune cells such as macrophages (Shutov et al., 2016). However, the therapeutic potential of anti-NGF treatments (or TrkA inhibitors) in the treatment of pain is more established (Da Silva et al., 2019). Additionally, whether EREG-mediated signaling cascades occur in primary afferents, immune cells, or both, remains to be elucidated.

##### Mechanistic Target of Rapamycin Complex

The mammalian target of rapamycin (mTOR) controls cellular metabolism and many other cellular functions by incorporating diverse signals from ligand-binding to intracellular cues regarding energy and nutrient availability (Liu and Sabatini, 2020). In mammals, mTOR functions as the catalytic subunit of at least two complexes (C1 and C2) that are distinct in their composition, activation, and cellular functions, which have been reviewed elsewhere (Kim and Guan, 2019; Liu and Sabatini, 2020). Importantly, mTORC1 is activated by PI3K-Akt signaling and mediates some critical aspects of PI3K-Akt signaling (Manning and Toker, 2017).

Given the activation of mTORC1 downstream of PI3K-Akt signaling, this kinase may mediate some of the important pain signals driven by EGFR and/or PI3K. Consistent with this, mTORC1 inhibitors such as rapamycin (also known as sirolimus) and rapamycin derivatives such as temsirolimus (CCI-779) inhibit mTORC1 and have been used in clinical trials for cancer therapy (Zhou and Huang, 2010; Dreyling et al., 2016; ). Administration of rapamycin (or another inhibitor of PI3K and mTORC1, PI-103) reduces nociceptive behaviors in the late phase of the formalin test (Xu et al., 2011). In male mice, local (intraplantar) or systemic (intraperitoneal) administration of temsirolimus attenuates SNI- and carrageenan-induced mechanical hypersensitivity, and SNI-induced cold hypersensitivity in the acetone test. However, local or systemic administration of temsirolimus treatment does not block SNI- or carrageenan-induced heat hypersensitivity, suggesting mTORC1 may contribute to SNI-induced mechanical and heat hypersensitivity, but not cold hypersensitivity (Obara et al., 2011).

Similarly, temsirolimus and rapamycin block EREG-mediated hypersensitivity in the formalin test (Martin et al., 2017). Hence, while some evidence for mTORC1 exists in mediating pain signaling, given that rapamycin, and potentially its derivatives, may inhibit both mTOR complexes (Zhou and Huang, 2010), the mechanism(s) by which mTOR complexes may contribute to EREG mediated pain sensitization remains to be further examined.

##### Mitogen-Activated Protein Kinase

The family of mitogen-activated protein kinases (MAPKs) includes three major members: extracellular regulated kinase (ERK), p38 and c-jun N-terminal Kinase (JNK). Notably, EGFR signaling activates Erk in most cell types leading to proliferation and differentiation (Dong et al., 2004; Wee and Wang, 2017). p38 and JNK are activated in a cell and tissue-context-dependent manner, in which stresses such as tissue damage can lead to the activation of p38 and JNK signaling, in which both have roles in various programmed cell death pathways (Jin et al., 2003; Zhuang et al., 2005; Cuadrado and Nebreda, 2010; Lee et al., 2020). Following peripheral nerve injury, these MAPKs are differentially expressed in astrocytes and in microglia, important cellular players in the development of neuropathic pain (Jin et al., 2003; Zhuang et al., 2005; Zhuang et al., 2006). Different MAPKs are necessary for the development of distinct phases of neuropathic pain. For instance, the phosphorylation and activation of p38 and ERK in spinal microglia is necessary to establish SNL-induced nociceptive behaviors in mice (Jin et al., 2003; Zhuang et al., 2005), whereas JNK or ERK activation in spinal astrocytes is necessary for the development and maintenance of SNL-induced mechanical allodynia, which is blocked by inhibition of JNK (Ji et al., 2009; Zhuang et al., 2005; Zhuang et al., 2006). Nociceptive stimuli (mechanical, heat, cold) but not innocuous light touch induce ERK activation within the dorsal horn. Pre-treatment with MEK inhibitor PD 98059 blocks formalin-induced nocifensive and nociceptive behaviors in rats (Ji et al., 1999). Additionally, pre-administration of a p38 inhibitor prevents SNL-induced mechanical allodynia (Jin et al., 2003).

In terms of drug development, p38 MAPK is a novel target of cytokine-suppressive anti-inflammatory drugs due to its regulatory role in the synthesis of inflammatory intermediates (Anand et al., 2011). In the CFA model of inflammatory pain, CFA administration leads to activation of p38 and ERK1/2 in the DRG, and inhibition of p38 may reduce neuronal sensitization (Zhang et al., 2018). Additionally, chronic compression of the DRG induces mechanical allodynia that is attenuated following intrathecal injections of MAPK inhibitors in rats (Qu et al., 2016). In a clinical trial, patients with nerve trauma, radiculopathy or carpal tunnel syndrome, that were treated with dilmapimod, a p38 MAPK inhibitor, demonstrated a significant reduction in pain (Anand et al., 2011). However, larger clinical trials assessing dilmapimod use are needed for clinically meaningful effect sizes and diversity of cohorts to demonstrate the analgesic effects of inhibiting p38 MAPK (Anand et al., 2011). While these studies collectively indicate that p38 MAPK, Erk and JNK may each contribute to pain, whether and how EGFR modulation or pain requires these signals remains to be examined.

### Indirect Actions of Epidermal Growth Factor Receptor Through Crosstalk With Signaling by Other Receptors

Recent evidence has shown that EGFR has non-canonical roles in regulating physiological processes such as inflammation, and pathophysiological functions in inflammatory pain and neuropathic pain. Though we have discussed how EGFR may regulate pain through direct ligand-induced activation of EGFR and canonical downstream signaling intermediates, EGFR also controls cellular processes indirectly through crosstalk with other plasma membrane receptors. Ligand-bound EGFR may induce the activation of another receptor or another receptor may transactivate EGFR. Here, we focus on two mechanisms by which EGFR regulates pain-generating processes through receptor crosstalk.

#### Epidermal Growth Factor Receptor Crosstalk: Toll-Like Receptor 4

Toll-like receptors (TLRs) have key roles in the activation of the innate immune system by detecting pathogen-associated molecular patterns (PAMPs) and damage-associated molecular patterns (DAMPs) associated with various microbes and cellular damage, respectively (Kawasaki and Kawai, 2014). Upon binding their respective PAMPs or DAMPs, active TLRs mediate immune responses and cytokine production (Kawasaki and Kawai, 2014). TLR activation in various cell types, including microglia, astrocytes, and sensory neurons, has been implicated in the development of persistent pain [reviewed by Lacagnina et al. (2018)]. Among the most widely studied of these is TLR4, which makes up part of the extracellular lipopolysaccharide (LPS) receptor. Lipopolysaccharides (endotoxins) are a major constituent of the gram-negative bacterial outer membrane that mediate various inflammatory signaling pathways (Mazgaeen and Gurung, 2020). In the context of pain signaling, LPS stimulation may sensitize TRPV1-positive neurons within trigeminal ganglia by sensitizing capsaicin-induced inward calcium currents and release of calcitonin gene-related peptide, a pain mediator (Diogenes et al., 2011). Furthermore, LPS may contribute to pain sensitization mediated by other triggers, as seen for example by blockage of paclitaxel-induced capsaicin sensitization in DRG and spinal dorsal horn neuron by administration of the TLR4 antagonist, LPS-RS (Li et al., 2015).

While EGF may be sufficient to induce microglial chemotaxis (Nolte et al., 1997), this may involve cross-talk with LPS and/or TLR4 signaling, as EGFR can also affect the LPS-induced chemotaxis of primary microglia. Inhibition of EGFR with AG1478 blocks LPS-induced chemotaxis of primary microglia (Qu et al., 2015). Moreover, inhibition of EGFR in primary microglia (Qu et al., 2012), and inhibition or shRNA silencing of EGFR in bone marrow-derived macrophages (BMDM) (Chattopadhyay et al., 2015), blocks LPS-induced production of proinflammatory cytokines IL-1β and TNFα *in vitro*. A similar result has been reported in bone marrow derived macrophages and RAW 264.7 cells, in which inhibition of EGFR reduces LPS-stimulated IL-1β, IL-6, IL-10 and TNFα expression (Tang et al., 2020), indicating that EGFR is essential for LPS-induced regulation of inflammation in macrophages.

While TLR4 activation may stimulate EGFR activation to elicit changes in inflammatory signaling, other studies suggest that EGFR may also act as an upstream mediator of TLR4 signaling, perhaps as part of a reciprocal activation mechanism involving EGFR and TLR4. In human mammary epithelial cells, EGF stimulation induces TLR4 phosphorylation and inhibition of EGFR with erlotinib blocks EGF-stimulated TLR4 phosphorylation, suggesting EGFR stimulation alone is sufficient to induce TLR4 activation (De et al., 2015). siRNA silencing of TLR4 does not block EGF-stimulated EGFR phosphorylation (Y1068) (De et al., 2015), suggesting that EGFR may act as an upstream mediator of TLR4 signaling. In support of this notion in an *in vivo* LPS instillation model of endotoxemia, EGFR inhibition improves animal survival in part by attenuating IL-6, TNFα and CXCL1 upregulation (De et al., 2015). Additionally, in a rat SCI model, inhibition of EGFR prevents the transcritpional upregulation of IL-1β and TNFα, further highlighting the ability of EGFR to modulate pro-inflammatory signaling (Qu et al., 2012).

**FIGURE 4:**
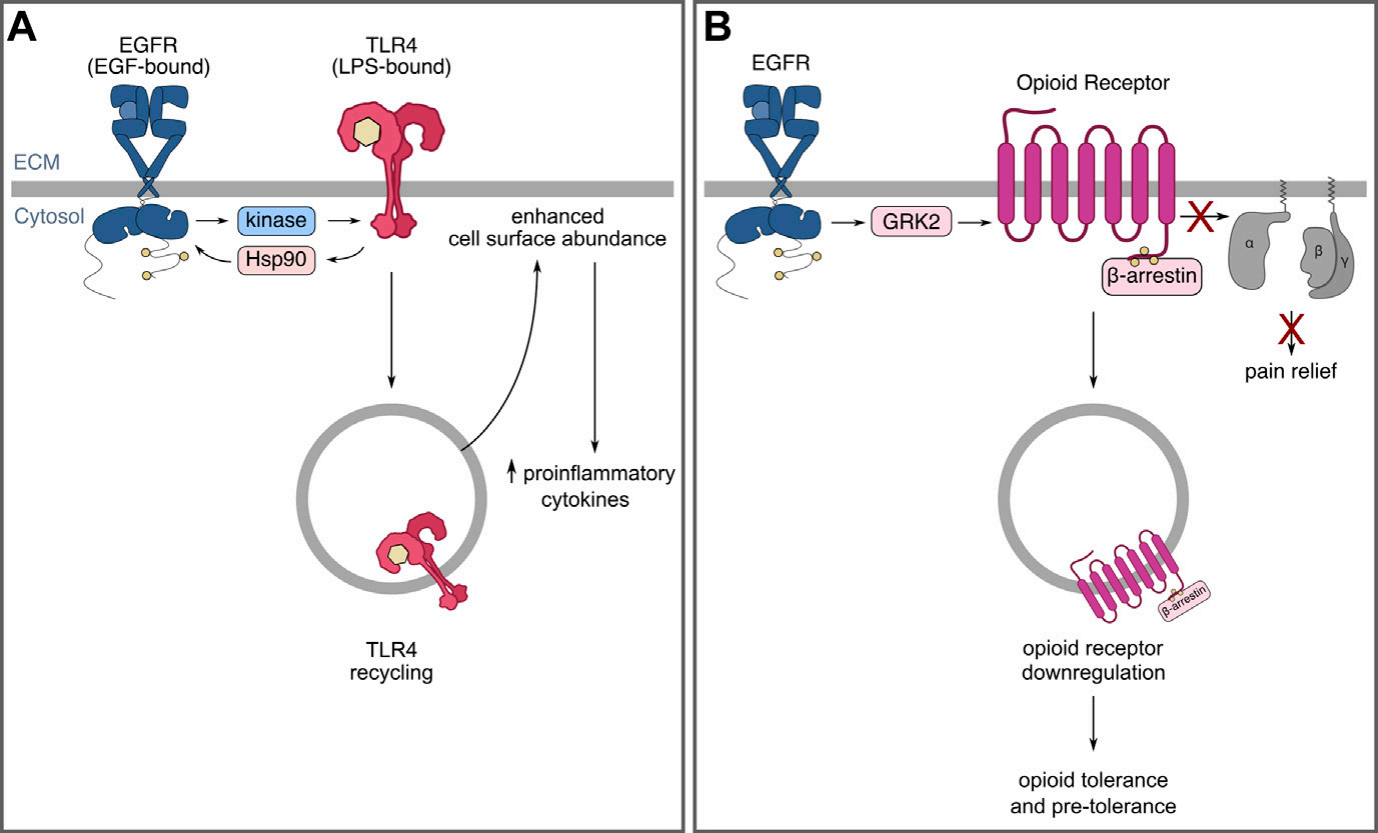
EGFR may modulate immune responses and opioid tolerance by controlling receptor localization and receptor crosstalk. **(A)** EGFR is required for aspects of TLR4 signalling as well as TLR4 recycling and cell-surface enrichment following LPS stimulation. **(B)** EGFR and opioid receptor crosstalk leading to opioid receptor internalization and downregulation that may contribute to tolerance.

The mechanisms by which EGFR may be required for TLR4 signaling have been recently investigated. Some evidence suggests that TLR4 may transactivate EGFR through protein intermediates, such as Hsp90 (Thuringer et al., 2011), while other evidence suggests that the Src family kinase, Lyn, may be required for some pathways activated by EGFR-mediated TLR4 signalling (De et al., 2015). A recent study has shown that EGFR augments TLR4 cell surface abundance in murine bone marrow-derived macrophages by mediating TLR4 internalization and subsequent traffic that leads to TLR4 recycling (Tang et al., 2020), which may in turn enhance the magnitude or duration of TLR4 signaling. These results were not sex-dependent (Tang et al., 2020), unlike other neuroimmune mechanisms of pain (Mapplebeck et al., 2018). Collectively, these data present evidence for mechanisms by which EGFR may control signalling by receptors involved in inflammatory responses, such as TLR4, and that this regulation may be part of positive feedback linking activation of EGFR and TLR4 in select cells such as macrophages (Figure 4A). Future studies should be directed at investigating the functional implications of the extensive crosstalk between TLR and EGFR signaling pathways on pain sensitization and chronic neuropathic and non-neuropathic pain.

#### Epidermal Growth Factor Receptor Crosstalk: Opioid Receptors

Opioids continue to be a mainstay of pain management; however preclinical and clinical evidence suggests that continued use may contribute to neural changes that promote the transition from acute to chronic pain [reviewed in Kandasamy and Price (2015)]. The “hyperalgesic priming” model developed by Jon Levine and colleagues describes that following the resolution of an acute “priming” stimulus (e.g., carrageenan), a secondary stimulus (that is normally subthreshold) leads to a prolonged state of hypersensitivity (Kandasamy and Price, 2015). One such secondary stimulus is prostaglandin E2 (PGE_2_), which drives sensitization of peripheral nerve terminals and is a major cause of inflammatory pain (Scholze et al., 2014). Opioids may induce the prolongation or maintenance of PGE_2-_induced hyperalgesia (Araldi et al., 2015), and some evidence has shown that EGFR may be required for this process as well. DAMGO, a mu-opioid receptor agonist, prolongs PGE_2_-induced hyperalgesia. Importantly, DAMGO-induced hyperalgesia prolongation is blocked by inhibition of EGFR (Araldi et al., 2018), suggesting that EGFR is required for mu-opioid receptor-mediated prolongation of PGE_2_-induced hyperalgesia. This evidence demonstrates that EGFR may have roles in the mechanisms by which opioid receptors prolong states of hypersensitivity.

EGFR may also participate in driving opioid tolerance through opioid receptor downregulation (Figure 4B). Opioids have widely been reported to have low analgesic efficacy in patients and animal models of neuropathic pain, which may be, in part, due to their downregulation in neuropathic pain (Obara et al., 2007). Opioid receptors of mu (MOR), delta (DOR), and kappa (KOR) subtypes, are G-protein coupled receptors (GPCRs) (Allouche et al., 2014) expressed within the nervous system, including the DRG (Zhou et al., 2014; Sun et al., 2017) and dorsal horn (Ikoma et al., 2007; Zhou et al., 2014). Upon ligand-binding, the active GPCR induces not only canonical G-protein signaling, but also the activation of GPCR kinases (GRKs) that promote the association of β-arrestins to the receptor, thereby inducing receptor internalization and eventual down-regulation (Allouche et al., 2014). Accumulating evidence demonstrates that various opioid receptors are down-regulated in various animal models of neuropathic pain (Obara et al., 2007).

Crosstalk between EGFR and G-protein-coupled receptors (GPCRs), including that between EGFR and opioid receptors (Belcheva et al., 2003; Chen et al., 2008; Clayton et al., 2010; Lennon et al., 2014), has been established in various contexts and may result in EGFR-driven downregulation of opioid receptors. EGF pre-treatment promotes agonist-induced DOR and MOR internalization, and EGF stimulation alone promotes GRK2 traffic to the plasma membrane, EGFR association with GRK2, and GRK2 phosphorylation *in vitro* (Chen et al., 2008). Similarly, EGFR may attenuate G-protein signaling and lead to the downregulation of other GPCRs through GRK2 phosphorylation, such as the dopamine D2-like receptor, D_3_R (Sun et al., 2018). Since opioid receptor agonists induce receptor internalization that may lead to their downregulation, it has been proposed that opioid receptor downregulation in neuropathic pain is due to the disease itself, rather than medication (agonist)-induced downregulation (Sun et al., 2017). However, as these studies indicate, EGFR-dependent modulation of opioid receptors may have a central role in pain and in opioid tolerance.

Consistent with a possible role of EGFR in modulating opioid receptor signaling, clinical case reports indicate that pharmacological inhibition of EGFR may have effects on opioid efficacy and tolerance. In the case series conducted by Kersten and colleagues, some patients were able to decrease their opioid dose significantly within days following treatment with cetuximab (Kersten et al., 2013). In addition, these patients experienced significant pain relief for the first time in months and experienced improvements in physical functioning. This notion has been supported by preclinical evidence, in which inhibition of EGFR (gefitinib) reverses morphine tolerance in rats following SNL by restoring the analgesic effect of morphine against mechanical allodynia (Puig et al., 2020). This suggests that therapeutics targeting EGFR may be repurposed to restore the analgesic effect of morphine in neuropathic pain, though this should be replicated with larger and more diverse cohorts to determine whether the generalizability of these findings. In this case, EGFR inhibitors may provide both direct analgesic effects in addition to improving the pharmacologic benefit and safety of opioids in patients with neuropathic pain.

The above is analogous to observations made in a rat CFA model, where inhibition of NGF-TrkA signaling reversed morphine tolerance (Mousa et al., 2007). In the SNL model, PDGFR inhibition similarly restores morphine analgesic effects in male rats (Donica et al., 2014) and inhibition of PDGFR-β reverses morphine tolerance, but does not eliminate the acute analgesic effect of morphine in rats (Wang et al., 2012). Administration of PDGFR agonist (PDGFR-BB) induces mechanical allodynia, which is blocked by inhibition of EGFR with gefitinib (Puig et al., 2020). However, whether PDGFR and EGFR crosstalk in driving opioid tolerance remains to be determined. Thus, while RTK signaling may drive opioid tolerance, the contribution of various RTKs such as EGFR in specific contexts warrants further investigation. Although studies by Puig et al. (EGFR) and Donica et al. (PDGFR) observed that administration of gefitinib or imatinib, respectively, do not reverse SNL-induced mechanical allodynia, treatment with these respective inhibitors restores the analgesic effect of morphine in rats. Importantly, whether EGFR may drive opioid tolerance through peripheral or central mechanisms is incompletely understood.

## Concluding Remarks

Chronic pain, particularly chronic neuropathic pain, remains a global health priority due to a paucity of effective and well tolerated therapies. Inhibitors of EGFR may exert analgesic effects in patients with neuropathic pain with minimal side effects. These empiric clinical findings are supported by various preclinical models of pain hypersensitivity. However, the use of EGFR inhibitors in neuropathic pain treatment may be cost-prohibitive, with costs per month in the thousands of dollars (Wang et al., 2013; Yang et al., 2020). Additionally, preclinical studies demonstrating roles of EGFR in pathological non-neuropathic pain are limited, and whether inhibitors of EGFR exert analgesic effects in patients with pathological, non-neuropathic pain remains to be determined. Considerable work remains in both the clinical and preclinical spheres to evaluate the benefit of repurposing EGFR inhibitors for patient care and understanding the mechanistic basis for EGFR’s involvement in pain.

The mechanisms by which EGFR may mediate or contribute to the initiation and maintenance of neuropathic pain are likely diverse given the pleiotropic cellular functions of this receptor family. These may include both direct actions of the receptor by binding its cognate ligands and indirect actions through transactivation. Few studies have investigated the signaling mechanisms by which EREG may mediate sensitization, though it appears that similar to NGF-mediated sensitization, this mechanism requires PI3K. Thus, an outstanding and fundamental question surrounds whether RTKs have shared mechanisms by which they impact pain hypersensitivity or have distinct pathways. The latter is important as the differential contributions and molecular pathways may inform pharmacologic strategies with increased context specificity. Current evidence suggests that class I PI3K are the main contributors in neuropathic pain. Future work directed at delineating the specific roles of specific class I isoforms would be beneficial since there are a variety of pan- and isoform-specific inhibitors in clinical trial. Importantly, there are other pain mediators, such as STAT3 (Liu et al., 2013; Zhang et al., 2017; Hu et al., 2020), that are also activated by EGFR (Han and Lo, 2012). The extent to which these targets contribute to EGFR-mediated hypersensitivity remains to be elucidated.

Limitations of animal models of pain [reviewed by Mogil (2009)] are influenced by many factors such as methodological differences, strain and genotype, social interactions, and sex affect various aspects of pain physiology and pathophysiology (Deuis et al., 2017). Sex differences in neuroimmunology and pain perception [reviewed by Mapplebeck et al. (2016), Rosen et al. (2017)] and in the expression of EGFR, its ligands and EGFR signalling (Treister et al., 2005; Liu et al., 2016; Zhang et al., 2019) are well documented. Yet, many clinical and pre-clinical studies provide little data around sex and gender as a variable in their studies. As a result, whether sex differences exist in the mechanisms by which EGFR mediates pain, and the therapeutic implications of such differences, remains to be elucidated.

EGFR may influence pain directly or indirectly by controlling traffic of other pain-associated proteins, such as TRPV1 or TLR4 and opioid receptors, respectively. Given the importance of neuro-immune interactions in pain, the roles EGFR in these processes warrant further investigation. The reduced effectiveness of opioids and increased tolerance in patients with neuropathic pain remains problematic. As such, the preclinical evidence suggesting that EGFR inhibition may reverse morphine tolerance is exciting and requires further evaluation.

The evidence we reviewed here positions EGFR as an important player in pathological pain states and provides the impetus for the clinical and biomedical research communities to investigate EGFR as a potential therapeutic target.
